# Misleading Lesions in Gynecological Malignancies: A Case Report of Desmoid Tumor During Pregnancy and a Narrative Review of the Literature

**DOI:** 10.3390/jcm14217815

**Published:** 2025-11-03

**Authors:** Emma Bonetti Palermo, Federico Ferrari, Cecilia Dell’Avalle, Ilaria Nodari, Emma Paola Ongarini, Iacopo Ghini, Andrea Giannini, Hooman Soleymani majd, Giuseppe Ciravolo, Franco Odicino

**Affiliations:** 1 S.C. Ostetricia e Ginecologia, ASST Spedali Civili Brescia, Dipartimento Area Della Donna e Materno Infantile, 25136 Brescia, Italy; 2 Department of Clinical and Experimental Sciences, University of Brescia, 25136 Brescia, Italy; c.dellavalle@unibs.it (C.D.); nodilaria@gmail.com (I.N.);; 3 Gynaecology Oncology, Oxford University Hospitals NHS Foundation Trust, Oxford OX3 7LE, UK; 4 Department of Pathology, ASST Spedali Civili Brescia, 25136 Brescia, Italy; 5 Department of Gynecological, Obstetrical and Urological Sciences, “Sapienza” University of Rome, Italy-Policlinico Umberto I, Viale Regina Elena, 328, 00161 Rome, Italy

**Keywords:** desmoid tumor, desmoid-type fibromatosis, abdominal wall, pregnancy, abdominal-wall endometriosis, differential diagnosis

## Abstract

**Background**: Desmoid tumors (DTs) are rare, locally aggressive soft-tissue neoplasms that often affect women of reproductive age. Pregnancy and prior abdominal surgery or trauma have been associated with tumor development and growth, while imaging frequently overlaps with abdominal-wall endometriosis. We present the case of a 39-year-old woman with an abdominal-wall DT and provide a narrative review of the literature focused on pregnancy/postpartum patterns, differential diagnosis, and management. **Methods**: A narrative review of PubMed/MEDLINE and Web of Science (January 1982–December 2024) was conducted. We included English-language case reports/series, narrative/descriptive reviews, and consensus statements relevant to DTs in pregnancy or reproductive-age women, emphasizing abdominal-wall disease. **Results**: The patient’s right abdominal-wall mass enlarged during pregnancy and further post-partum imaging repeatedly suggested endometriosis. En bloc resection revealed desmoid-type fibromatosis composed of bland spindle cells in a collagenous stroma, with nuclear β-catenin and lymphoid enhancer–binding factor 1 (LEF1) positivity on immunohistochemistry. Magnetic resonance imaging (MRI) at 12 months showed no recurrence. Across included studies, pregnancy and post-partum enlargement is common, abdominal-wall DTs frequently mimic scar endometriosis, and pre-operative ultrasound has limited specificity. Current practice supports watch-and-wait for stable, asymptomatic lesions and function-preserving surgery for symptomatic progression, while systemic options (anti-estrogens, low-dose chemotherapy, and tyrosine kinase inhibitors) are reserved for progressive or unresectable disease. Recurrence risk relates to age, size, site, and β-catenin status; future pregnancy is not contraindicated. **Conclusions**: Abdominal-wall DTs, although rare, should be considered in the differential diagnosis of reproductive-age women presenting with abdominal-wall masses, particularly during or after pregnancy.

## 1. Introduction

Desmoid tumors (DTs), also called desmoid-type fibromatosis (DF) or aggressive fibromatosis, are rare, benign, soft-tissue neoplasms characterized by monoclonal myofibroblastic proliferation, infiltrative growth, and a high propensity for local recurrence despite a lack of metastatic potential [1,2,3]. DTs represent less than 3% of all soft-tissue tumors, with an annual incidence of 5–6 cases per million population [4], and they predominantly affect women of reproductive age [5].

Pathogenesis is heterogeneous. Somatic activating mutations in the CTNNB1 gene (Wnt/β-catenin pathway) are the predominant driver in sporadic DTs, whereas germline mutations in the APC (adenomatous polyposis coli) gene underline FAP (familial adenomatous polyposis) and Gardner syndrome [6,7]. Estrogenic stimulation appears to modulate tumor behavior, as suggested by the female predominance, growth during or shortly after pregnancy, and occasional regression after menopause or anti-estrogen therapy [8,9].

Reported risk factors also include prior abdominal trauma or surgery, including laparotomy, laparoscopy, or cesarean section (C-section) scars. Environmental factors such as chronic mechanical stress and local inflammatory signaling are also thought to influence tumor initiation and subsequent growth [10].

Clinical courses are highly variable and location dependent [11]. Anatomically, DTs are classified as extra-abdominal (43%) or abdominal (57%). Abdominal DTs are further subdivided into abdominal-wall lesions (49% of all DTs) and intra-abdominal lesions (8%). Abdominal-wall lesions typically originate within the rectus sheath, whereas intra-abdominal tumors are most frequently mesenteric and may entangle bowel loops [7,12].

Ultrasound (US), computed tomography (CT), and magnetic resonance imaging (MRI) guide diagnosis and surgical planning, but definitive diagnosis is histopathological [13,14,15]. Historically, complete surgical excision was the standard of care, with systemic therapy or radiotherapy reserved for unresectable or recurrent disease [8]. Contemporary algorithms favor an initial “watch-and-wait” strategy because 20–30% of DTs—especially those of the abdominal wall—undergo spontaneous regression, and post-resection recurrence rates remain high (18–56%) [6,16]. Refining predictors of aggressive behavior or recurrence remains a research priority. Management is further complicated during pregnancy, a not-infrequent context given the tumor’s demographic profile and hormonal sensitivity [12].

In this article, we report the case of a 39-year-old woman with an abdominal-wall desmoid tumor diagnosed during pregnancy and complement it with a narrative literature review. By combining the clinical course with published evidence, we highlight diagnostic challenges, the influence of pregnancy-related hormonal changes, and evolving management strategies, aiming to improve understanding of this rare condition.

## 2. Case Report

A 39-year-old Italian woman (born 1985) presented at our tertiary care center (University of Brescia, Italy) with a slow-growing, painless mass of the right abdominal wall. Her medical history included hyperhomocysteinemia and three spontaneous miscarriages. Previous surgery comprised open splenectomy after traumatic rupture (2000). 

The patient first attended our Endometriosis Clinic in 2017. Transvaginal US showed a 32 mm unilocular, ground-glass cyst in the left ovary, interpreted as an endometrioma. Pelvic MRI in 2020 confirmed the endometrioma, rectovaginal septal thickening, and bilateral uterosacral-ligament (USL) involvement, supporting diagnosis of endometriosis. After hysteroscopic removal of an endometrial polyp (September 2020), the patient conceived via in vitro fertilization (IVF) and delivered vaginally in 2021 without complications. 

**Figure 1:**
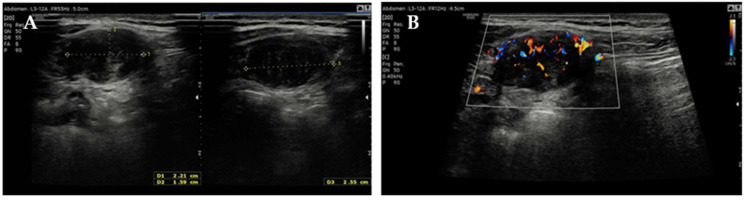
Transabdominal US of the right abdominal wall: (**A**) measurements of the lesion and (**B**) its color-Doppler vascularization.

Follow-up MRI (May 2023) performed elsewhere revealed a solid, ovoid, hypointense nodule located between the right internal oblique and transversus abdominis muscles, described as consistent with fibrotic sequelae of endometriosis. Transabdominal US performed at our Clinic, confirmed a well-circumscribed, hypoechoic, moderately vascular lesion measuring 22 × 16 × 25 mm (Figure 1). Planned excision was postponed when the patient conceived again through IVF.

At 25 + 5 weeks’ gestation, US showed interval growth to 50 × 21 mm. Two weeks later the patient was admitted with acute abdominal pain. Because of suspected bowel obstruction, contrast-enhanced CT was performed and revealed an adhesion-induced volvulus of the first jejunal loop and a well-defined 50 × 30 mm infra-fascial right abdominal-wall mass. This lesion showed low baseline density (neither hemorrhagic nor adipose) with post-contrast enhancement. Multiple intraperitoneal nodules, compatible with post-splenectomy splenosis, were also noted (17 mm in the left flank, 74 × 20 mm in the mesogastrium above the uterus, and 20 mm in the left paramedian region with central calcification).

At 28 + 1 weeks’ gestation (February 2024) the patient underwent midline xipho-umbilical laparotomy with adhesiolysis, detorsion, and excision of the largest omental implant. Histology confirmed splenosis. The remainder of the pregnancy was uneventful. Elective C-section at 38 + 6 weeks (May 2024) via Pfannenstiel incision revealed no visible abdominal-wall masses.

**Figure 2:**
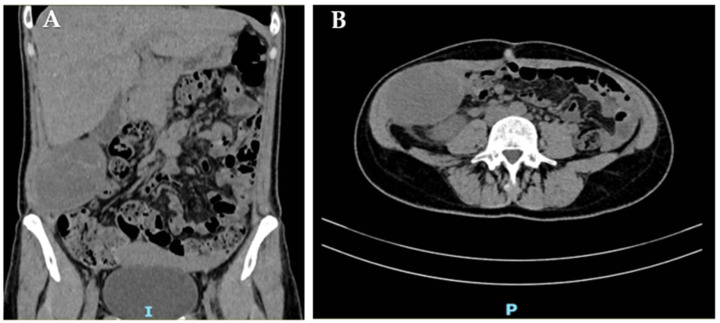
CT scan of the right abdominal wall: coronal (**A**) and axial (**B**) view of the abdominal-wall DT.

Two months post-partum, CT undertaken elsewhere for febrile pyelonephritis demonstrated marked enlargement of the known abdominal-wall lesion to 70 × 50 × 93 mm, displacing but not invading the right kidney (Figure 2).

The mass remained heterogeneous and predominantly hypodense with peripheral enhancement, and it was again interpreted as an endometrioma; residual splenosis nodules persisted (largest 3.3 cm).

**Figure 3:**
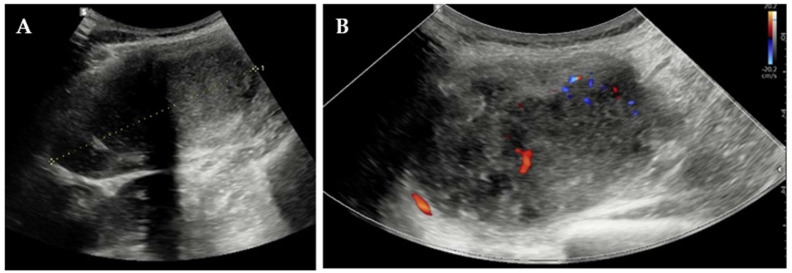
Pre-operative transabdominal US: (**A**) measurement of the lesion and (**B**) color-Doppler vascularization.

Elective excision was scheduled for August 2024 at our Clinic. Pre-operative blood tests, including complete blood count, hepatic and renal panels, and tumor markers, were within normal ranges, except for a mildly elevated α-fetoprotein level (8.5 ng/mL; reference < 5.8 ng/mL) while the patient was breastfeeding. Pre-operative US depicted an encapsulated, heterogeneous, moderately vascular mass measuring 86 mm (Figure 3) and suggested either endometriosis or splenosis.

**Figure 4:**
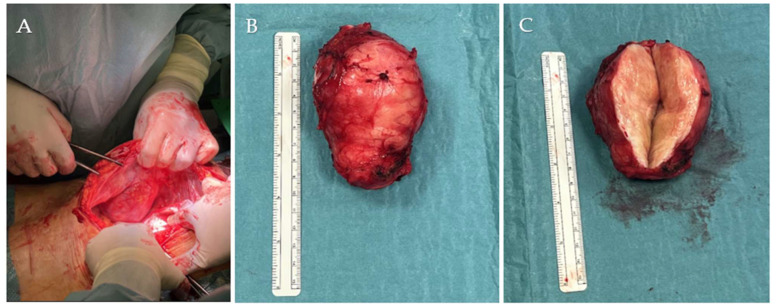
Macroscopic tumor appearance: (**A**) lesion in situ during resection and (**B**) gross specimen after excision and (**C**) after cutting the surface.

Through re-opening of the previous midline incision, an encapsulated 9 cm tumor situated between the abdominal musculature and parietal peritoneum was excised en bloc (Figure 4A). Macroscopically, it was characterized by a firm texture, with a smooth surface and a whitish color; on section it appeared fibrous, woody, and lardaceous (Figure 4B,C).

**Figure 5:**
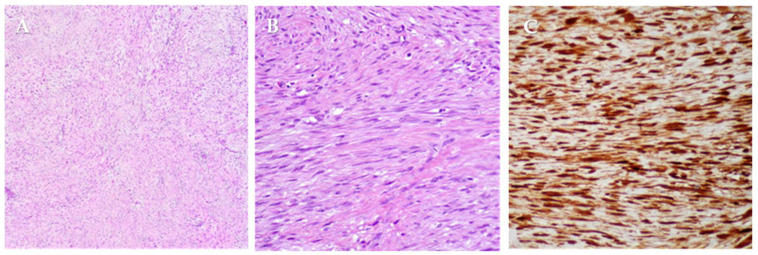
Histopathological and immunohistochemical features of desmoid-type fibromatosis. (**A**) Low-power view (hematoxylin and eosin staining) shows a proliferation of uniform spindle cells arranged in long and intersecting fascicles dispersed in a dense collagenous stroma. (**B**) Higher magnification highlights spindle cells with elongated nuclei, displaying bland cytologic appearance. (**C**) Immunohistochemical staining for β-catenin reveals both strong cytoplasmic and nuclear positivity in neoplastic cells.

Histology demonstrated desmoid-type fibromatosis, characterized by fascicular proliferation of cytologically bland spindle cells within a dense collagenous stroma, with occasional mitotic figures and scattered mast cells. Peripheral infiltration of skeletal muscle was observed. Immunohistochemistry showed β-catenin and LEF1 (lymphoid enhancer-binding factor 1) positivity, focal desmin staining, and negativity for α-smooth-muscle actin, diffuse desmin, S-100, SOX10, broad-spectrum cytokeratins (MNF-116), and CD34 (Figure 5).

Surveillance MRI every six months remains negative for recurrence 12 months post-operatively (August 2025).

## 3. Materials and Methods

We conducted a narrative review using PubMed/MEDLINE and Web of Science, including publications from 1 January 1982 to 31 December 2024. The search combined controlled vocabulary (MeSH in PubMed) and free-text terms. Search terms included: “desmoid tumor”, “desmoid-type fibromatosis”, and “aggressive fibromatosis” combined with “pregnancy”, “post-partum”/“puerperium”, “hormonal status”, “abdominal wall” and “endometriosis”.

We included English-language articles—case reports and case series, narrative/descriptive reviews, and consensus statements—relevant to desmoid tumors in pregnancy or in women of reproductive age, with emphasis on differential diagnosis with abdominal-wall endometriosis and on tumor behavior during pregnancy and the puerperium. We excluded non-English papers, conference abstracts without sufficient data, editorials, and letters lacking primary clinical information.

This case report adheres to the CARE guidelines (EQUATOR Network). Written informed consent was obtained from the patient. Ethical approval was not applicable.

## 4. Results

Our narrative review comprised 18 manuscripts focusing specifically on abdominal-wall desmoid tumors. Of these, 16 case reports and 2 case series, encompassing a total of 22 patients, specifically addressed desmoid tumors in pregnant or post-partum women or in the context of oral-contraceptive exposure; additional narrative/descriptive reviews and consensus statements informed background and management. Study-level details are provided in Supplementary Table S1.

### 4.1. Epidemiology

DT is a rare condition, accounting for less than 3% of all soft-tissue tumors and 0.03% of all neoplasms, with an incidence of 5–6 cases per million/year [2,17,18]. It is more common among female patients (more than twice F > M), especially young women in their reproductive age with a peak of diagnosis between the 3rd and 4th decade of life [19]. Pregnancy or the post-partum period is reported at diagnosis in 11–32% of women [20]. Additionally, most of the abdominal forms of the disease occur in women in their reproductive age (20–40 years old) [8].

### 4.2. Etiology

Most DTs are sporadic, while a minority are associated with FAP [21]. Tumorigenesis is linked to dysregulation of the APC/Wnt–β-catenin pathway in myofibroblasts [6]. In most cases this reflects somatic, stabilizing mutations of CTNNB1 (β-catenin; 85–90%), which promote nuclear β-catenin accumulation and transcriptional activation; 10–15% of cases are instead related to germline APC mutations (FAP/Gardner syndrome). Accordingly, detection of a CTNNB1 mutation supports a sporadic origin, whereas an APC germline alteration and/or a polyposis phenotype suggests a hereditary form. Histologically the two groups are indistinguishable, but their molecular backgrounds differ [1,7].

### 4.3. Risk Factors

Multiple factors have been implicated in DT development [22]: hormonal influences are suggested by presentation during or shortly after pregnancy, association with oral contraceptives, female predominance in reproductive age, occasional regression at menopause, estrogen β receptor expression, and reports of shrinkage with anti-estrogen therapy [23].

Trauma/surgery is another recognized factor: prior abdominal incisions—laparotomy, laparoscopy, or C-section scars—are linked to DTs in both sporadic and FAP-related disease, including during pregnancy [19,24], likely via aberrant wound healing with sustained myofibroblast activation and matrix deposition [25]. These signals align with the abdominal-wall predilection in reproductive-age women. More than half of the patients (12 out of 22) had a history of previous abdominal surgery, most commonly C-section (Supplementary Table S1).

### 4.4. Histological and Immunohistochemical Factors

Macroscopically, DTs are firm, gray-whitish masses with a scar-like appearance [8]. Microscopically, they comprise bland spindle myofibroblasts arranged in long fascicles within a collagen-rich stroma; they are typically poorly circumscribed and infiltrative, entrapping adjacent soft tissues [8,26]. Mitotic activity is low, with no significant atypia, necrosis, or vascular invasion. Nuclei are small and vesicular; peripheral inflammatory cells (e.g., macrophages, occasional giant cells, and lymphocytes) may be present, and keloid-like hyalinization can be seen in some lesions [27]. Histology does not distinguish sporadic from FAP-associated DTs, although their molecular profiles differ.

Immunohistochemistry supports the diagnosis: tumor cells are vimentin-positive, show focal/patchy SMA reactivity, and are generally negative for desmin, cytokeratins, and S-100. Nuclear β-catenin accumulation is highly suggestive of desmoid-type fibromatosis [27].

### 4.5. Diagnosis

A careful family history is essential to screen for a familial form (FAP): relatives with multiple colonic polyps or colectomy—especially with childhood onset—raise suspicion [1]. Personal history should document sex, reproductive/hormonal status, and prior surgery/trauma [11].

Clinical presentation depends on site and size. Intra-abdominal DTs may remain silent or present with pain/obstruction when large [8]. By contrast, abdominal-wall DTs—often arising from the rectus or internal oblique and their fascia—typically present as painless, firm, subfascial masses and are detected earlier because they are palpable [28]. Paresthesias may occur with nerve compression [19].

Definitive diagnosis is histopathological, but imaging is pivotal for characterization and planning. US is a common first test for abdominal-wall masses: DTs show variable echogenicity, oval shape, and smooth to ill-defined margins; color-Doppler flow is likewise variable [15,29]. On contrast-enhanced CT, DTs appear as soft-tissue masses with well-defined margins (classically abdominal wall) or ill-defined margins (intra/extra-abdominal). Attenuation is similar/slightly higher than muscle with mild-to-moderate enhancement, reflecting collagen and myxoid content [26,29]. MRI typically shows isointense T1 and iso- to hyperintense T2 signals with heterogeneous enhancement; lower T2 correlates with dense collagen/hypocellularity, whereas higher T2 suggests greater cellularity [29,30]. Enhancement degree varies and relates to collagen deposition [6].

For follow-up, CT is useful to track size change, while MRI better delineates extent (including vascular relationships) and detects post-operative recurrence [8].

### 4.6. Differential Diagnosis

Abdominal-wall masses may reflect infection, endometriosis, inflammatory lesions, neoplasms, or hematoma [26]. Imaging findings often overlap across entities, so features are rarely pathognomonic. Histologic differentials for desmoid tumors include fibrosarcoma, solitary fibrous tumor, dermatofibrosarcoma protuberans, and reactive fibrous proliferations (scar, hypertrophic scar/keloid), as well as nodular fasciitis [8]. Immunohistochemistry aids distinction, particularly when imaging is non-specific [8]. Lesion size, site, and clinical context (e.g., reproductive age, prior surgery/trauma) should be integrated into the diagnostic assessment [27].

### 4.7. Treatment

Desmoid tumors are locally infiltrative and may compromise adjacent structures; although non-metastatic, they can cause substantial morbidity through mass effect and obstruction [27]. Management is not always straightforward. Decisions balance early detection, the role/timing of surgery versus non-operative options (like radiotherapy and systemic therapies), and the tumor’s variable natural history—some lesions remain stable or regress spontaneously—so watch-and-wait is appropriate in selected cases [8,12]. Observation is reasonable for small, asymptomatic lesions without threat to vital structures, with clinical and imaging follow-up (e.g., CT/MRI) to track behavior [1].

According to consensus-based guidelines from the National Comprehensive Cancer Network (NCCN), surgical resection is considered when technically feasible in symptomatic disease or where there is imminent risk to surrounding organs [31]. As reported in Supplementary Table S1, surgical resection was performed in 18 cases (82%). A multimodal approach may include radiotherapy and systemic therapy [1]. Radiotherapy is effective for unresectable tumors or poor surgical candidates but is used mainly for extra-abdominal sites and rarely for abdominal/intra-abdominal disease due to enteritis risk [1,8].

Systemic therapy is typically reserved for progressive/unresectable tumors or multiple recurrences to induce remission and prevent complications [8]. Options include NSAIDs and anti-estrogen agents (e.g., tamoxifen); responses can occur even without demonstrable ER expression, but efficacy is variable and potential adverse effects require caution [17,32]. Cytotoxic chemotherapy may be used both as a neo-adjuvant option (to decrease tumor’s size before surgery) and when non-cytotoxic options fail for large inoperable tumors [8,32]. Evidence of clinical benefit and patient’s tolerance are the main factors determining the duration of these treatments: regimens of methotrexate and vinblastine or doxorubicine and dacarbazine show activity, though nausea/vomiting and late cardiotoxicity (with doxorubicin) must be considered [8].

Among tyrosine kinase inhibitors (TKIs), sorafenib is active in progressive desmoid tumors but is teratogenic [1]. Pazopanib, approved for soft-tissue sarcomas, has shown activity in retrospective series of desmoid tumors [1,33].

### 4.8. Natural History and Risk of Recurrence

The natural history of DTs is heterogeneous: spontaneous regression has been reported (e.g., at menopause or after bilateral oophorectomy); some lesions recur or progress despite treatment; and up to 50% may remain stable [34]. During pregnancy and post-partum, enlargement occurs in at least one-third of cases [35]. Among the 22 patients reviewed (Supplementary Table S1), tumor enlargement during pregnancy or postpartum was reported in 11 cases.

After resection of abdominal-wall DTs, reported recurrence rates are 40–45% [36,37]. The risk of local recurrence depends on different factors. Historically, positive margins correlated with higher local recurrence [38]. However, as pointed out by the ESMO Group 2017 revision on DT, patient age, tumor size and site, and β-catenin status should be considered in estimating the risk of local recurrence; resection should aim for negative margins, but not at the expense of function [6].

Most recurrences arise within 2–3 years, supporting the importance of close imaging follow-up [8]. If recurrence occurs, options include repeat surgery and non-operative therapies, tailored to symptoms and risk. Future pregnancy is not contraindicated [1].

## 5. Discussion

The World Health Organization (WHO) defines desmoid-type fibromatosis as a “clonal proliferation that arises in the deep soft tissues and is characterized by infiltrative growth and a tendency to local recurrence, but an inability to metastasize” [3]. Although rare (5–6/million/year), DTs are relatively frequent in women of reproductive age, which fits with our patient [1].

In pregnancy, the abdominal wall is the most frequently affected site [39,40]. Etiology is heterogeneous: sporadic tumors with aberrant β-catenin signaling predominate in young women, while associations with Gardner syndrome/FAP are also reported [12,41,42]. Moreover, in both cases (sporadic and familial forms) personal history of previous C-sections or abdominal traumas is quite a common finding; Robinson et al. proposed that gestational stretching of muscles and fascia may favor tumor development, while growth in other locations during and after pregnancy points to a contribution of hormonal and immunological changes [43]. Similarly, Carneiro et al. suggested occult microtrauma from aponeurotic strain or even fetal movements as an additional trigger [44]. The hormonal status linked to pregnancy and post-partum can accelerate growth. In a multicenter series by Fiore et al., at least one-third of DTs enlarged during gestation or shortly after delivery [35].

Several reports illustrate substantial growth during pregnancy. Michopoulou et al. described a 37-year-old in whom an abdominal-wall DT enlarged from 3 × 2 cm at 16 weeks to 20 × 16 cm at 38 weeks, when C-section was performed [45]. Similarly, Mohd-Sulaimain et al. reported a 20-year-old with a left rectus abdominis tumor detected at 13 weeks that reached 15 × 12 cm by 34 weeks, prompting elective C-section [41]. Zhou et al. documented an extreme case: 35 × 30 × 14 cm (7.1 kg) at C-section after measuring 15 × 12 cm at 21 weeks, while Viriyaroj et al. reported 28 × 21 × 18 cm (5 kg) at delivery [46,47].

Post-partum enlargement is also described: Gurluler et al. observed growth from 10 × 5 cm to 26 × 12 × 6.5 cm in a right rectus lesion after delivery [48]. Conversely, small lesions also occur: Way and Culham reported a 1.3 cm abdominal-wall tumor six months after C-section, and Carneiro et al. described a 2 cm lesion 4 weeks postpartum [44,49].

Diagnosis is histological and supported by immunohistochemistry: bland spindle myofibroblasts in collagenous stroma with nuclear β-catenin, variably positive for vimentin and SMA, and negative for desmin, cytokeratins and S-100—features mirrored in our case. Emerging data implicate the PD-1/PD-L1 axis, correlating with β-catenin expression and immune markers like CD4, CD8, and IFN-γ, potentially affecting prognosis [50].

In practice, core-needle biopsy is not consistently performed for DTs during pregnancy or the post-partum period; in our review, only 11 of 22 cases had pre-operative histological confirmation (Supplementary Table S1) [12,16,41,44,45,48,49,51,52,53,54], and imaging often guides management.

Ultrasound is the first-line imaging test but is not specific; its reported preoperative accuracy is approximately 50% [25,55]. The modest pre-operative accuracy of ultrasound underscores the need to complement it with cross-sectional imaging and, when safe, histological confirmation, to reduce misclassification and avoid delays in management.

The differential diagnosis on ultrasound commonly includes fibroids, hematoma, and abdominal-wall endometriosis [56,57,58]. During the second and third trimester, uterine enlargement brings the uterus close to the abdominal wall and can obscure cleavage planes, further complicating assessment [56].

Post-partum abdominal-wall masses are often initially attributed to hematoma, especially after a recent C-section. In the case reported by Gurluler et al., the subfascial location and recent C-section supported this hypothesis; however, the absence of spontaneous regression and persistent symptoms prompted re-evaluation and an alternative diagnosis [48].

During or after pregnancy, abdominal-wall DTs, especially in the lower quadrants near a C-section scar, often mimic scar endometriosis. Clinical features are non-specific (endometriosis may be asymptomatic or present with vague pain), and palpation or ultrasound alone rarely distinguishes the two. Depth can help: DTs usually arise from the musculo-aponeurotic plane, whereas endometriotic implants more often lie in the subcutaneous tissues [59].

CT is a useful adjunct to define the lesion’s site and its relationships with adjacent tissues, but MRI is the preferred modality for diagnosis and follow-up of abdominal-wall DTs [28]. For abdominal-wall endometriosis, discriminating CT signs are limited; Yarmish et al. described the “gorgon sign”—linear strands radiating into subcutaneous fat from a central nodule below the umbilicus with a homogeneous appearance—as suggestive. On MRI, a T1-hyperintense focus that persists on fat-suppressed sequences also favors endometriosis [60]. Imaging must be interpreted with the clinical history, and both DT and abdominal-wall endometriosis can occur without prior surgery [61].

Management must consider clinical context as well as tumor site, size, and growth pattern [25]. For stable, asymptomatic, non-threatening lesions, a watch-and-wait strategy is first-line [31,62], whereas rapid growth and/or symptoms warrant intervention [31].

In a multicenter cohort of 92 women, Fiore et al. reported 52% resection, 43% observation, and 4% medical therapy; among those resected, 13% relapsed, while 14% of patients showed spontaneous regression, supporting conservative policies when feasible. After pregnancy, 46% underwent treatment and >50% were observed: 17% relapsed post-treatment, and 27% experienced progression with a subsequent pregnancy [35].

Debaudringhien et al. also noted an association between prior pregnancy and progression or relapse [11], whereas Cates et al. reported that pregnancy itself does not increase local-recurrence risk after resection [63].

Obstetric outcomes in Fiore et al. were notable mainly for a higher C-section rate, though vaginal delivery has been reported even with large tumors, particularly at intra-abdominal sites [35,40,64].

When surgery is required for progressive enlargement and/or symptoms, abdominal-wall DTs are usually amenable to safe resection, but each case should be planned with tumor size and potential functional and aesthetic consequences in mind [65]. The priority is preservation of function, rather than achieving negative margins at any cost. Positive margins are not uncommon; evidence suggests that prognosis relates more to patient age, tumor size/site, and β-catenin status than to margin status [20]. Consistently, Carneiro et al. reported no recurrences at two years despite positive margins in their series [44].

Counseling about recurrence should note that pregnancy does not appear to increase the risk of local recurrence [15]. However, in the absence of robust consensus guidance, close follow-up is advisable and a prudent interval of at least two years after treatment is recommended before attempting another pregnancy [23].

This report presents an abdominal-wall DT with pregnancy/post-partum evolution and prior surgery, highlighting the frequent mimicry with abdominal-wall endometriosis and the resulting diagnostic challenges—areas only partially addressed in previous narrative reviews and case reports [12,66,67]. This work is limited by its single-patient design and a narrative (non-systematic) review based on heterogeneous case reports and small series. Small numbers, possible publication bias toward large or endometriosis-mimicking tumors, language/database restrictions, non-uniform follow-up, and inconsistent imaging and molecular reporting preclude quantitative synthesis and constrain generalizability. Future work should prioritize prospective multicenter registries with standardized datasets and routine molecular/immune (CTNNB1/APC, PD-1/PD-L1) profiling, correlating biomarkers with growth, recurrence, and response to watch-and-wait, surgery, or systemic therapy to refine risk stratification and individualize care as happening for endometrial and ovarian cancer.

## 6. Conclusions

Abdominal-wall DTs are uncommon but should not be overlooked in women of reproductive age, particularly during or after pregnancy. Their rarity and non-specific imaging features can delay diagnosis, so clinicians should keep them in the differential when assessing abdominal-wall masses in this setting. Management has shifted from routine radical surgery to a watch-and-wait approach for stable, asymptomatic lesions, reserving surgery for symptomatic progression or threatened function. Further studies are needed to refine radiological and immunohistochemical predictors of behavior and recurrence, enabling more precise treatment selection and individualized follow-up strategies.

## Data Availability

No new data were created or analyzed in this study. Data sharing is not applicable to this article.

## Supplementary Materials

The following supporting information can be downloaded at: https://www.mdpi.com/article/10.3390/jcm14217815/s1, Table S1: An overview on selected abdominal wall desmoid tumor cases in pregnant/post-partum and fertile women, showing clinical behaviors, therapeutic approaches and follow-up features, specifically eventual recurrences and subsequent pregnancies. (LPS: laparoscopy; FAP: familial adenomatous polyposis; CS: c-section; VD: vaginal delivery; SA: spontaneous abortion; N.A.: not applicable; CHT: chemotherapy; CNB: core needle biopsy; GWs: gestational weeks).

## Abbreviations

The following abbreviations are used in this manuscript:

APC	adenomatous polyposis coli
CIN3	cervical intraepithelial neoplasia 3
CT	computed tomography
C-section	cesarean section
DF	desmoid fibromatosis
DT	desmoid tumor
ER	estrogen receptor
ESMO	European Society for Medical Oncology
F	female
FAP	familial adenomatous polyposis
IFN-γ	interferon gamma
IVF	in vitro fertilization
LEF1	lymphoid enhancer–binding factor 1
M	male
MRI	magnetic resonance imaging
NCCN	National Comprehensive Cancer Network
NSAIDs	nonsteroidal anti-inflammatory drugs
PD-1	programmed cell death protein 1
PD-L1	programmed death-ligand 1
SOX10	SRY-box transcription factor 10
TKIs	tyrosine kinase inhibitors
US	ultrasound
USL	uterosacral ligament
WHO	World Health Organization
