# Supplementary material for: Misleading Lesions in Gynecological Malignancies: A Case Report of Desmoid Tumor During Pregnancy and a Narrative Review of the Literature
Source: J Clin Med. 2025 Nov 3;14(21):7815. doi: 10.3390/jcm14217815 (PMC12608541; doi:10.3390/jcm14217815)
Supplement: Supplementary file 1 [file jcm-14-07815-s001.zip › jcm-3890128-supplementary.pdf]

Supplementary Material

Table S1. An overview on selected abdominal wall desmoid tumor cases in pregnant/post-partum and fertile women, showing clinical behaviors, therapeutic approaches and follow-up features, specifically eventual recurrences and subsequent pregnancies. (LPS: laparoscopy; FAP: familial adenomatous polyposis; CS: c-section; VD: vaginal delivery; SA: spontaneous abortion; N.A.: not applicable; CHT: chemotherapy; CNB: core needle biopsy; GWs: gestational weeks).

Paper	Age (y)	Parity	First discovery of an abdominal wall mass	Previous Surgeries	Volumetric increase during pregnancy/post-partum	Type of delivery (if pregnant)	Definitive diagnosis on CNB	Treatment	Surgical margins	Recurrences	Other pregnancies
Zubor et al, 2024 [12]	29	Nullipara	3 years before pregnancy	LPS total proctocolectomy for FAP syndrome	N.A.	N.A.	Yes	CHT	N.A.	Yes, during pregnancies	Yes, 1 induced medical abortion at 6 weeks; subsequent other pregnancy with CS at 38 weeks
Fujita et al, 2023 [16]	30	1 CS	10-weeks pregnant	1 CS	Yes, from 5x4 cm at diagnosis to 9 cm at surgery	CS at 38 GWs	Yes	Surgery during the 17th gestational week	Positive	No	Unknown
Mohd Sulaiman et al., 2022 [41]	20	1 CS	13-weeks pregnant	1 CS	Yes, from 4x7x10 cm at diagnosis to 15x12 cm at surgery	CS at 34 GWs	Yes	Surgery	Unknown	Unknown	No
Zhou et al, 2015 [46]	31	1 VD	21-weeks pregnant	None	Yes, from 15x12 cm at diagnosis to 35x30x14 cm at surgery	CS at 35 GWs	No	Surgery	Unknown	No	Unknown
Vural et al, 2015 [14]	42	1 CS	10 years after pregnancy	1 CS	N.A.	N.A.	No	Surgery	Negative	No	No
Leon et al, 2015 [15]	24	1 SA, 2 VD)	2 years before pregnancy	None	Yes, from 11.7x12.3x17 cm at 14-weeks pregnant to 25 cm at C-section	CS at 38 GWs	No, differential diagnosis with uterine fibroid	None (intra-op evaluation during CS was made with the decision not to intervene)	N.A.	Unknown	Unknown
Gurluler et al, 2014 [48]	35	1 VD, 1 CS	3 weeks after C-section	1 CS	Yes, from 5 cm at diagnosis to	N.A.	Initial differential diagnosis with	Surgery	Negative	Unknown	Unknown

					26x12x6.5 cm at surgery		hematoma, then definitive diagnosis via tru-cut biopsy				
Awwad et al, 2013 [56]	40	1 SA, 1 CS	20-weeks pregnant	1 CS	Yes, up tp 12x9.5x7 cm at surgery	CS at 39 GWs	No, differential diagnosis with uterine fibroid	Surgery	Negative	No	Yes, 1 subsequent pregnancy after 3 years, delivered via CS
Krentel et al, 2012 [59]	25	1 CS	38-weeks pregnant	1 CS	Unknown, tumor's dimensions at surgery: 3 cm	CS at 38 GWs	No, differential diagnosis with scar endometriosis	Surgery	Negative	No	Unknown
Choi et al, 2012 [67]	36	2 VD	3 years after last VD	None	N.A.	N.A.	No	Surgery	Negative	No	Unknown
Durkin et al, 2011 [53]	29	1 VD	1st trimester of pregnancy	None	Yes, from 3.5x7.2 cm at diagnosis to 18.5x15x9 cm at surgery	VD at 39 GWs	Yes	Surgery during 22nd week of gestation	Negative	No	No
Michopoulou et al, 2010 [45]	37	1 CS	1st trimester of pregnancy	1 CS	Yes, from 3x2 cm at diagnosis to 20x16 cm at surgery	CS at 38 GWs	Yes	Surgery during post-partum	Unknown	Unknown	Unknown
Viriyaroi et al, 2009 [47]	17	1 VD	2nd trimester of pregnancy	None	Yes, from 20x20 cm at diagnosis to 28x21x18 cm at surgery	CS	No	Surgery during post-partum	Unknown	No	Unknown
Le Roc'h et al, 2009 [51]	18	Nullipara	2 years before pregnancy	None	Yes, up to 8.7x4.8 cm at 39 GWs	VD	Yes	None	Unknown	Unknown	Unknown
Carneiro et al, 2008 [44] 4 cases in a case series	36	1 CS	13 months after CS	1 CS	No	N.A.	No	Surgery	Positive	No	Unknown
	39	3 CS	1 month post-partum	3 CS, 1 myomectomy	No	N.A.	No, differential diagnosis with hernia	Surgery	Unknown	No	Unknown

	32	1 VD	12 months post-partum	None	No	N.A.	Yes, before CNB differential diagnosis with hematoma	NSAIDs, Tamoxifen, Imatinib and surgery	Negative	No	Unknown
	39	1 CS	12 months post-partum	1 CS, 1 myomectomy, breast cancer, thyroidectomy for thyroid cancer	No	N.A.	No	Surgery	Negative	No	Unknown
De Cian et al, 1999 [54]	42	1 CS	12 weeks pregnant	1 CS	No	CS at 37 GWs	Yes	Surgery	Positive	No	Unknown
Way and Culham et al, 1997 [49] 2 cases in a case series	28	2 VD	Within first month of postpartum	None	No	N.A.	Yes	Surgery	Negative	No	Yes, 3 subsequent pregnancies (2 abortions, 1 uncomplicated VD)
	28	1 VD	12 months after VD	None	No	N.A.	Yes	Surgery	Negative	No	Yes, 1 subsequent pregnancy with VD
Camiel and Solish et al, 1982 [68]	22	1 VD	3rd trimester	None	Yes	VD	Yes (after delivery)	Surgery	Positive	Unknown	Unknown
